# Effectiveness of Cognitive Behaviour Therapy for Mothers of Children with Food Allergy: A Case Series

**DOI:** 10.3390/healthcare3041194

**Published:** 2015-11-25

**Authors:** Rebecca C. Knibb

**Affiliations:** Department of Psychology, School of Life and Health Sciences, Aston University, Birmingham B4 7ET, UK; E-Mail: r.knibb@aston.ac.uk; Tel.: +44-0121-204-3402; Fax: +44-0121-204-3696

**Keywords:** anxiety, CBT, food allergy, parents, quality of life, worry

## Abstract

*Background:* Food allergy affects quality of life in patients and parents and mothers report high levels of anxiety and stress. Cognitive Behaviour Therapy (CBT) may be helpful in reducing the psychological impact of food allergy. The aim of this study was to examine the appropriateness and effectiveness of CBT to improve psychological outcomes in parents of children with food allergy. *Methods:* Five parents (all mothers) from a local allergy clinic requested to have CBT; six mothers acted as controls and completed questionnaires only. CBT was individual and face-to face and lasted 12 weeks. All participants completed measures of anxiety and depression, worry, stress, general mental health, generic and food allergy specific quality of life at baseline and at 12 weeks. *Results:* Anxiety, depression and worry in the CBT group significantly reduced and overall mental health and QoL significantly improved from baseline to 12 weeks (all *p* < 0.05) in mothers in the CBT group; control group scores remained stable. *Conclusions:* CBT appears to be appropriate and effective in mothers of children with food allergy and a larger randomised control trial now needs to be conducted. Ways in which aspects of CBT can be incorporated into allergy clinic visits need investigation.

## 1. Introduction

Food allergy is an immune-mediated adverse reaction to ingestion of food protein, which causes unpleasant symptoms including rash, itching, swelling of mouth and lips, vomiting and anaphylaxis which is a systemic reaction which can be life threatening if not promptly treated with adrenaline [1]. Prevalence rates are increasing and currently around 5%–8% of children are affected [1,2]. Treatment involves avoidance of the implicated foods and emergency treatment of symptoms caused by accidental ingestion. Constant vigilance is therefore needed to avoid these symptoms and a number of studies have found that food allergy can have a big impact on the quality of life (QoL) of children and adult sufferers as well as their immediate family [3]. Food allergy has also been found to be associated with higher levels of stress and anxiety in parents, particular mothers, caring for a food allergic child, compared to a general healthy population [4,5]. It is thought that the constant vigilance needed to check the safety of foods for their child and the anxiety caused by the potential fatal consequences of accidental ingestion of an allergen are to blame [4].

Qualitative work has supported these findings and also provides further insights into why food allergy can cause such distress. Akeson *et al.* [6] explored the psychosocial impact of living with anaphylaxis in seven parents of children with food allergy. Parents had vivid recall of severe reactions in their children, which was seen as a traumatic experience and could explain why they developed high levels of anxiety levels which persisted long after the incident. Parents also exhibited great anxiety in handing responsibility over to their child (such as reading own food labels, doing own cooking) and wanted to retain the control of the food allergy themselves. In a study of six mothers of children aged 6 to twelve years who were at risk of anaphylaxis, a feeling of living with risk was predominant [7]. The risks were perceived not just from the allergy, but from the environment and from people around them who might not understand food allergy. Mothers also felt that they lived in fear for both the present and the future for their child.

Looking after a child with food allergy therefore has an effect on the mental health status of the parent and guidelines on the management of food allergy state that reducing distress in parents is the next important step [8]. Information about food allergy and advice from health care practitioners at allergy clinics does not seem to help reduce levels of distress [9] and so for some families further support is warranted. Group interventions may be helpful; LeBovidge *et al.* [10] delivered an educational workshop to parents and children with food allergy, which resulted in parents reporting an increase in perceived competence in coping and reduction in burden of coping with their child’s allergy. The study did not use validated measures or a control group and so although results provide some preliminary evidence for the efficacy of educational group interventions, further research is needed. Polloni *et al.* [11] assessed reasons for psychological treatment for food allergy in 100 consecutive families attending an allergy clinic in Italy. A total of 40% reported emotional and social problems such as anxiety, fear, stress, social isolation and poor self-esteem. A further 40% reported difficulties in managing food allergy and poor coping strategies for dietary management. Psychological support was provided mostly for children and adolescents by a clinical psychologist, although there is a lack of detail provided regarding the type of support offered. All patients reported their problems were a bit or much better after receiving support and nearly all said their problems were now more bearable.

For many families then, brief interventions may help them manage food allergy more effectively and reduce the stress and worry that can be involved. In some cases though, parents suffer prolonged and very high levels of anxiety and worry, particularly if the child has suffered a life threatening anaphylactic reaction [6,7]; for these parents more targeted therapy such as individual Cognitive Behaviour Therapy (CBT) may be more effective. CBT is an evidence based talking therapy which focuses on present difficulties including symptoms, emotions, behaviour and negative thoughts. Therapy involves an assessment of how these factors interact to cause and maintain mental health issues such as anxiety and depression and techniques are used to change behaviour or thoughts to help promote better mental health. It has been shown to be extremely effective for reducing levels of anxiety (e.g., [12,13]) and effective for people suffering from chronic conditions such as asthma and chronic obstructive pulmonary disease (e.g., [14,15]). Randomised control trials for CBT for those with asthma has been found to increase QoL and reduce anxiety [14,16], reduce depression and asthma-specific fear and improve asthma specific QoL [17]. These studies adopted individual face-to-face CBT over eight to ten weeks offering support for this method of CBT delivery in allergy related conditions.

Very few studies have investigated efficacy of CBT for improving psychological outcomes in parents. Wong and Poon [18] provided ten weeks of group CBT to 58 Chinese parents of children with developmental disabilities and found significant improvements in general health of the parent, parental stress and parental quality of life compared to a control group post-treatment. No study to date has reported on the appropriateness or effectiveness of individual face-to-face CBT for improving psychosocial outcomes in parents of children with food allergy. The presented case series aims to investigate whether individual CBT is an appropriate therapy for these parents and whether it could be effective in reducing anxiety, depression, worry and stress and improve QoL.

## 2. Methods

### 2.1. Design

This study employed a non-randomised case control design and a case series of five parents who underwent CBT is presented here. The study was given ethical approval by the Derby Hospitals NHS Trust Research Ethics Committee for the East Midlands (reference number: 11/EM/0401). It was also registered with the Derbyshire NHS Trust Research and Development Office. All participants were recruited from Derby Children’s Hospital and gave written informed consent before taking part in the study.

### 2.2. Participants

Participants (*n* = 11) were all mothers of children diagnosed with food allergy at a local allergy clinic; 5 mothers had CBT and 6 mothers were recruited to a control group.

#### 2.2.1. Inclusion Criteria

Participants had to have at least one child with a clinical diagnosis of food allergy (diagnosed by skin prick tests, blood tests or food challenge by an allergy specialist or paediatrician). Participants requesting CBT had to have difficulties that were suitable for treatment with this therapy; this included high stress levels, high anxiety, high levels of worry and/or low mood. This was assessed during a CBT assessment session and was aided by the scores obtained on the questionnaires, and the diagnostic manual DSM-IV-R [19]. Participants also needed to be able to understand written and spoken English.

#### 2.2.2. Exclusion Criteria

Exclusion criteria were currently being treated by a psychiatrist or a psychotherapist; taking medication for anxiety or depression; currently suffering from a chronic illness that may impact on quality of life or mental health; not having a child that suffers from food allergy.

### 2.3. Materials

#### 2.3.1. Demographic and Food Allergy Questionnaire

A questionnaire to gather demographic information from the parent and food allergy information about their child was developed based on that used in previous published studies [5]. Information collected included the type of food allergy, symptoms, how the allergy was diagnosed, medication, history of anaphylaxis and presence of other atopic conditions such as asthma, hay-fever and eczema.

#### 2.3.2. Hospital Anxiety and Depression Scale (HADS)

The HADS [20] is a 14-item self-report measure of anxiety and depression; higher scores indicate greater anxiety and depression. It has cut off scores to detect clinical cases of anxiety and depression: scores of 0–7 are classified as normal, 8–10 as mild, 11–14 as moderate and 15–21 as severe. The measure has excellent internal consistency with Cronbach’s α of 0.93 for anxiety and 0.90 for depression and the scale has good construct validity [21].

#### 2.3.3. Perceived Stress Scale (PSS-14)

The PSS-14 [22] is a 14-item self-report measure which asks about thoughts and feelings in the last month, with a higher score indicating greater perceived stress. This measure has good internal consistency (Cronbach’s α = 0.84) [23].

#### 2.3.4. Food Allergy Quality of Life-Parental Burden Scale (FAQL-PB)

The FAQL-PB scale [24] is a 17-item instrument which asks about the burden of food allergy felt by the family; higher scores indicate greater perceived burden. Internal consistency in a U.K. sample [25] has been reported as strong (Cronbach’s α = 0.95) and it also demonstrated good convergent and construct validity.

#### 2.3.5. The World Health Organisation QoL Scale (Brief Version) (WHOQOL-BREF)

The WHOQOL-BREF [26] is a 26 item QoL scale, which measures four major domains: physical, psychological, social relationships and environment. A higher score on this scale relates to better quality of life. The scale has good reliability across domains (alphas range from 0.68 to 0.82) [26].

#### 2.3.6. Penn State Worry Questionnaire (PSWQ)

The PSWQ [27] is a 16 item scale to measure worry. Higher scores indicate greater worry, with scores over 45 indicating Generalised Anxiety Disorder. The scale has excellent internal consistency (Cronbach’s α > 0.7), good test re-test reliability and good validity [27].

#### 2.3.7. General Health Questionnaire-12 (GHQ-12)

The GHQ-12 [28] is a 12 item scale of current mental health. Scores over 11–12 indicate clinical caseness. The scale has excellent internal consistency (Cronbach’s α = 0.77–0.93) and good validity [28].

#### 2.3.8. Problems and Targets

A problem list and end targets were developed collaboratively with each participant following established guidelines [29]. Problem statements were based on areas mothers wanted to address where they felt they were not coping well and were causing emotional distress. They were rated on a 9 point Likert Scale from 0 to 8 for how much the behaviour interfered with their normal activities (behaviour) (0 = does not to 8 = continuously) and for how much the problem upset them (discomfort) (0 = does not to 8 = very severely). Targets for each problem statement were developed and similarly rated on a 9 point scale from 0 to 8 for how often they did the target behaviour (0 = complete success to 8 = have not made a start) and how much discomfort the target behaviour caused them (0 = none to 8 = very severe).

### 2.4. Procedure

Parents of children who had been diagnosed with food allergy by a local allergy clinic were given information about the study, either in person when they attended the clinic, or by a letter sent to their home from the clinic. Parents interested in having CBT were invited to a full assessment session. Parents not interested in having CBT sessions but wanted to take part in the study were asked to act as controls and complete questionnaires only.

Participants requesting CBT were seen on an individual basis in one of the counseling rooms at the University or in their own homes. In the first session participants completed all questionnaires and a cognitive and behavioural functional analysis (which focuses on thoughts, behaviours, emotions and physical symptoms) was conducted in order to ensure they met the inclusion/exclusion criteria and to facilitate the development of an appropriate and tailored treatment plan for that individual [30]. The course of CBT was provided one-to-one, once a week for 12 weeks and each session lasted approximately one to one and a half hours. Problems and end targets were set with each individual and an evidence-based treatment plan was developed. Problems and targets were rated at regular intervals and all questionnaires were completed again at week 12. CBT was provided by a chartered and practitioner psychologist with extensive training (to Masters level) in CBT and in depth knowledge of food allergy (RK).

The control group completed the same questionnaires as the CBT group at baseline (during a face-to-face session with the researcher) and at week 12 (via post). No participants withdrew from the study, but 2 controls were lost to follow-up.

### 2.5. Data Analysis

All data was coded and analysed using SPSS version 20. All analyses were two-tailed with alpha set at 0.05. Kolmogorov-Smirnov tests were run to assess normality, which revealed most scale scores were not normally distributed, due to the low numbers of participants. Non-parametric tests were therefore used. Differences between cases and controls were analysed using Mann-Whitney U tests for Independent Samples. Changes in scale scores from baseline to end of therapy were analysed by Wilcoxon signed-rank tests. Effect sizes were estimated by converting z scores for each test statistic to *r* values (by dividing the *z* value by the square root of the number of observations); small effect sizes have r values of 0.1 to 0.3, medium effect sizes have *r* values 0.3–0.5, large effects are over 0.5 [31].

Participants did not have the same number of problems and targets, so baseline and end of treatment mean scores were calculated for each participant; all baseline problem scores were summed for each participant and divided by the number of problems set to derive a mean baseline problem score and the same was done for end of treatment problem scores. The same was applied to target scores. Differences in mean problem and target scores from baseline to end of intervention were analysed using Wilcoxon signed-rank tests. Improvement scores for problems and targets were calculated by taking the final mean score for success on a problem or target away from the initial mean score; larger scores indicated bigger improvements in reducing problems and attaining targets.

## 3. Results

### 3.1. Demographic and Food Allergy Details of Parents and Children

Demographic and food allergy details for parents in the CBT and control groups and their children can be seen in Table 1. The types of foods and symptoms were broadly similar for children of cases and controls. The notable differences were more egg and tree nut allergy, multiple food allergies and history of anaphylaxis in children of parents who had CBT, however, numbers were too low to run analysis to check for differences. There were no significant differences between the age of parents in the CBT group and control group or the age of children in these two groups (all *p* > 0.05) (Table 1).

**Table 1:** Demographic and food allergy details for parents and children in the CBT and control group.

Parent and Child Characteristics	CBT Group	Control Group
**Parents (n)**	5	6
Sex (n%) female	5 (100.0)	6 (100)
Ethnic origin (n%) White British	5 (100)	6 (100)
Age, mean in years (SD)	38.80 (4.38)	33.00 (8.46)
**Food Allergic Child**		
Sex (n%) male	3 (60.0)	3 (50.0)
female	2 (40.0)	3 (50.0)
Age, mean in years (SD)	6.60 (3.36)	5.00 (3.58)
Age range in years	2–9	2–12
Time since diagnosis, mean in years (SD)	5.67 (3.23)	3.87 (2.59)
**Food Allergy, n (%)**		
Peanut	3 (60.0)	4 (66.67)
Treenuts	3 (60.0)	2 (33.33)
Egg	5 (100.0)	0
Milk	3 (60.0)	3 (50.0)
Fish	0	0
Soy	0	2 (33.33)
Tomato	0	1 (16.67)
**Number of Food Allergies, n (%)**		
1 food	1 (20.0)	2 (33.33)
2 foods	1 (20.0)	3 (50.0)
3+ foods	3 (60.0)	1 (16.67)
**Type of Symptoms, n (%)**		
Respiratory	4 (80.0)	4 (66.67)
Swelling of face, lips, tongue, eyes	2 (40.0)	4 (66.67)
Gastrointestinal	5 (100.0)	5 (83.33)
Skin rash/hives	3 (60.0)	5 (83.33)
History of anaphylaxis	3 (60.0)	2 (33.33)
**Presence of Asthma, Hayfever, Eczema**	5 (100.0)	6 (100.0)
**Medication Prescribed, n (%)**		
Adrenalin auto-injector	3 (60.0)	2 (33.33)
Antihistamines	6 (100.0)	6 (100.0)

### 3.2. A CBT Model for Parents of Children with Food Allergy

**Figure 1:**
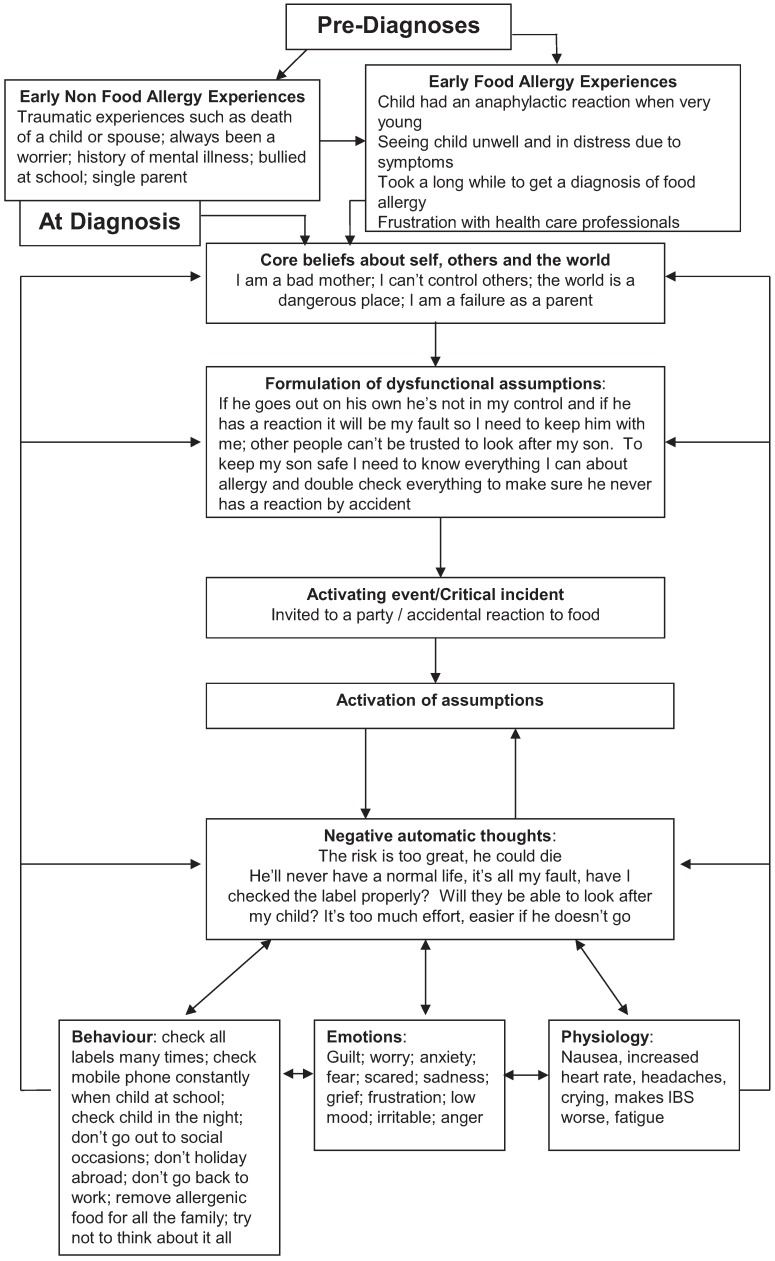
CBT model for food allergy.

After initial assessments had been conducted, a longitudinal idiographic model was developed which described the experiences of the participants and was used in subsequent sessions to aid therapy (Figure 1). The model included issues prior to the diagnosis of food allergy that parents thought affected their present difficulties. Some were related to food allergy, such as problems in getting a diagnosis for their child, and some were not, such as mental health difficulties prior to having a child with food allergy. The model also included core beliefs described by the participants as related to their child’s food allergy, such as being a bad mother, being a failure as a parent and that the world is an unsafe place.

Parents reported a number of dysfunctional assumptions they had developed, some examples of which can be seen in Figure 1. Activating events such as being invited to a party could trigger some or all of these dysfunctional assumptions and promote negative automatic thoughts which focused on risk, danger and inability of others to look after their child. A large list of emotions was generated by mothers, with some being more salient in particular situations. Anxiety was more predominant in situations such as being invited out to a social occasion or when trying a new food. Guilt was predominant when they had to ask someone else to look after their child or their child had an accidental reaction.

These thoughts and feelings were associated with avoidance behaviours, such as not eating out, not going on holiday abroad or simply trying not to think about issues such as the possibility of their child having an anaphylactic reaction in the future. Mothers also exhibited safety behaviours such as constant checking of their mobile phone when their child was not in their care. The physiology reported was similar to that exhibited in those suffering from anxiety or panic, such as increased heart rate; or was associated with low mood such as crying and fatigue. These thoughts, feelings, emotions and physiology then reinforced core beliefs and dysfunctional assumptions, thus maintaining the difficulties.

### 3.3. Problems and Targets and Interventions Used

Participants had between 3 and 4 problems each that they wished to address. Examples of these can be seen in Table 2. Each problem statement had a related end target and a range of evidence based CBT interventions were used to enable participants to achieve these. Examples of these can be seen in Table 3. For all participants psychoeducation about CBT, stress, anxiety, worry and depression was conducted. Education about food allergy was also delivered as confusion, misconceptions and erroneous beliefs were evident, particularly surrounding evidence for maternal diet causing allergy in their child, level of risk for child having a severe allergic reaction, how to use an adrenaline auto-injector (AAI), what skin prick tests showed and whether children can grow out of their allergies.

**Table 2:** Examples of problem statements from participants.

Problem Statements
I feel very anxious and cry when I think about what might happen if my son has a serious reaction as I feel as if I won’t know what to do and he might die, so I try not to think about it or talk about it.
I am very anxious when my son is not with me and I worry that he might eat something he shouldn’t and have a bad reaction, so I check my mobile every ten minutes in case I have missed a call about him, which makes me more anxious and I can’t concentrate on my work.
I worry about how my daughter will cope as she gets older and more independent and wants to go out on her own with her friends. I am scared she won’t know what to do if she has a bad reaction so I find it hard to let her have some control over what she eats.
I feel guilty asking other people to look after my daughter as they need to know what she can and cannot eat and how to give her an injection of adrenaline if she has a bad reaction, so most of the time I don’t let her go and stay with other people and that makes me feel guilty too.
When we are going through a bad time with my daughter’s eczema I don’t get a lot of sleep and during the day I can feel really frustrated and angry and I shout at my daughter, which makes me feel guilty as she gets upset.
I feel very anxious when we go out for the day as I have to do all the planning and work out what my son will be able to eat, I don’t get much help with this and I get frustrated and angry about that.

Baseline and end of intervention mean ratings and the mean change scores for each participant for frequency of behaviour and for discomfort caused can be seen in Table 4. There was a significant reduction in problem statement ratings from baseline to end of treatment for frequency of behaviour (*T* = 0; *z* = −2.03, *p* = 0.042, *r* = 0.64) and for discomfort (*T* = 0, *z* = −2.03, *p* = 0.042, *r* = .64). There was also a significant reduction in target statement ratings from baseline to end of treatment for frequency of behaviour (*T* = 0, *z* = −2.02, *p* = 0.043, *r* = 0.64) and for discomfort (*T* = 0, *z* = −2.02, *p* = 0.043, *r* = 0.64).

### 3.4. Impact of CBT on Quality of Life

At baseline cases had significantly poorer physical and psychological QoL than controls (*p* = 0.05). They also had significantly worse food allergy specific QoL (*p* = 0.004) (See supplementary Table S1 for the full data). At the end of the intervention there were no longer any significant differences between case and controls for QoL (all *p* > 0.05).

Participants in the CBT intervention group had significantly better physical QoL at the end of intervention compared to their baseline scores (*p* < 0.05). For all other generic areas of QoL the CBT group reported better QoL at the end of treatment but scores only approached significance. CBT participants had significantly less food allergy related parental burden at the end of the intervention (*p* < 0.05) (see supplementary Table S2). For controls there were no significant differences from baseline to the end of 12 weeks on any of these scale scores (all *p* > 0.05) showing that their QoL remained stable throughout the duration of the study (Supplementary Table S2). 

**Table 3:** Examples of end targets from participants with interventions utilised for each.

Target Statements	Interventions Used
I would like to feel less anxious about my son having a bad reaction and be more confident in knowing what to do if he does	1. Graded exposure to information about anaphylactic shock to reduce anxiety, including watching educational videos
	2. Psychoeducation about what might happen if a child goes into anaphylactic shock
	3. Role play using a trainer AAI with the parent and therapist
	4. Role play using a trainer AAI by the parent with their child
I would like to worry less about my child becoming more independent as he gets older and trust him to take more responsibility for his allergies	1. Worry tree to help decide what worries are controllable and what to do about them
	2. Behavioural experiment to see what happens if
	a. they allow child to read own food labels and decide what they can eat
	b. let child remember their medication and put their own creams on for their eczema
	3. Role play with child showing their friends how to use the trainer AAI in an emergency
I would like to be able to leave my child with people and not feel guilty about this, so that I have more time for myself, and worry less when he’s not with me	1. Psychoeducation and role play regarding how parent might teach someone else what they need to know about their child’s allergy and use of an AAI
	2. Behaviour experiment to test out consequences of leaving child with someone else
	3. Exploration of dysfunctional ways of thinking and making assumptions based on mind reading of others
I would like to be able to learn how to cope with things when my child is having a bad time with her eczema and not feel angry, upset or low	1. Diary to keep a log of mood
	2. Diary to log positive events and emotions in order to raise mood and self-esteem
	3. Challenging negative automatic thoughts such as “I cannot cope with this” and looking at evidence for and against such thoughts
I would like to look back on the time when my daughter was tiny and very ill with her allergy and not get upset about it and remember the good times	1. Emotional writing and re-living of past experiences to reduce grief associated with traumatic experiences
	2. Recording positive experiences from the past and the positive consequences that have occurred because of the food allergy, such as a better relationship with grandparents

**Table 4:** Mean problem and target baseline, end of treatment and change scores for each participant.

Participant	N of Problems	Baseline		End of Treatment		Change Score	
		Behaviour	Discomfort	Behaviour	Discomfort	Behaviour	Discomfort
1	4	7.0	7.5	4.0	4.5	3.0	3.0
2	4	5.5	6.25	1.5	2.25	4.0	4.0
3	3	7.3	8.0	3.7	3.3	3.6	4.7
4	3	6.0	8.0	2.0	2.0	4.0	6.0
5	4	7.5	7.5	2.75	3.5	4.75	4.0
Total		6.66	7.45	2.79	3.11	3.87	4.34
	**N of Targets**	**Baseline**		**End of Treatment**		**Change Score**	
		**Behaviour**	**Discomfort**	**Behaviour**	**Discomfort**	**Behaviour**	**Discomfort**
1	4	8.0	8.0	2.0	2.0	6.0	6.0
2	4	5.75	5.25	1.25	1.75	4.5	3.5
3	3	7.0	7.0	4.3	3.7	2.7	3.3
4	4	7.75	8.0	1.25	1.25	6.5	6.75
5	4	7.75	7.75	2.75	3.25	4.75	4.5
Total		7.25	7.20	2.31	2.39	4.89	4.81

### 3.5. Impact of CBT on Psychological Distress

At baseline the CBT group had significantly higher anxiety, depression and poorer general mental health (GHQ12 scores) than controls (all *p* < 0.05) (see supplementary Table S1). At the end of the intervention there were no longer any significant differences between cases and controls for anxiety and depression, however controls had significantly higher scores than cases on the GHQ12, demonstrating poorer mental health (*p* < 0.05) (see supplementary Table S1).

Participants in the CBT intervention group had significantly less worry, anxiety and depression at the end of the intervention compared to their baseline scores. They also had significantly lower scores on the GHQ12 indicating better mental health (all *p* < 0.05). Reductions in levels of stress only approached significance (see supplementary Table S2). For controls, there were no significant differences from baseline to the end of 12 weeks on any of these scale scores, showing stability over time for these variables.

At the start of the intervention four out of the five participants in the CBT group scored over the cut off levels for the GHQ12 and PSWQ and had moderate anxiety and three scored over the cut off for mild depression. Those scoring over the cut offs reduced over the course of the intervention, however numbers within each category were too small for analysis. (Full data can be seen in supplementary Table S3).

## 4. Discussion

This case series aimed to assess the appropriateness and effectiveness of Cognitive Behaviour Therapy for reducing psychological distress and improving quality of life in parents of children with food allergy. Mothers having CBT improved over time with significantly lower anxiety, depression and worry and better QoL at the end of therapy compared to their baseline scores. CBT therefore seems to be appropriate for this group of people.

### 4.1. A New CBT Model for Parents of Children with Food Allergy

The CBT model developed after assessments with the mothers identified important issues not reported in the food allergy literature before. Factors unrelated to food allergy; such as previous mental health difficulties; past traumatic experiences and being bullied at school are often present in people suffering from mental illness such as depression; panic and generalised anxiety disorder [32] but have not been identified as possible risk factors for parental distress in those with a child with food allergy. Similarly core beliefs such as I am a bad mother; it is my fault my child has an allergy and others cannot be trusted to look after my child have not been conceptualised as core beliefs in the food allergy literature. Other beliefs; such as seeing the world as unsafe; overestimating the risk of death from food allergy; feeling that others have no understanding of the seriousness of allergy and its consequences and believing they are a bad mother for restricting their child’s social life have been reported before (e.g., [7,33,34]). Further investigation is required to understand how these negative beliefs are formed; particularly concerning the time at which they develop and why some parents of children with food allergy have such beliefs and others do not. It may be that negative core beliefs not related to food allergy are prominent before diagnosis and they become more salient over time in parents who find it hard to adjust and cope with their child’s allergies. Experiences regarding their inability to cope may then reinforce such beliefs. Early detection of negative core beliefs is therefore extremely important in identifying parents in need of support.

Dysfunctional assumptions stemmed from mothers overestimating the risk of a serious reaction or death in their child and a need to keep their child safe. Although a level of vigilance is necessary and assessment of risk is a daily occurrence, participants in this study exhibited extreme levels of vigilance and avoidance. This could be due to previous experiences, particularly of anaphylaxis, increasing their fear, anxiety and uncertainty regarding the future health of their child. This level of vigilance can lead to more overprotective parenting styles [35], increase levels of anxiety [36] and prevent adaptive coping strategies from developing [33]. Negative automatic thoughts maintained these dysfunctional assumptions and triggered a range of emotions such as worry, anxiety, stress, fear, guilt, anger, sadness and grief. Anxiety and stress in particular have been identified in previous literature [4,9]. Anger, trauma and grief have only been reported very recently by parents who felt a loss of having a healthy child and the things they would not be able to do [33,37]. Experiences of an anaphylactic reaction in their child have been reported to have a lasting traumatic effect for the parent [6,37].

### 4.2. Psychological Outcomes for Parents of Food Allergic Children

Anxiety reported by mothers in the CBT group in the present study was mainly around fears of their child having a serious reaction and worry about using the adrenaline auto-injector. Proussel *et al.* [38] found that parental knowledge for use of an AAI is quite poor and even though parents are shown how to use an AAI in clinic it may be many years before they need to actually use one if their child has well managed allergy. Recent research has found that many mothers quickly forget how to use their AAI after training provided at an allergy clinic [39]. Role play with a trainer AAI worked extremely well in therapy in reducing anxiety and regular re-training in the use of AAIs would therefore be very useful, especially as parents who manage allergic reactions well can exhibit less distress [36]. Other anxieties were associated with letting others look after their child and their child becoming more independent as they grow older, which has been reported in the literature as an important issue to deal with [6,37].

Worry was very high in both the control and CBT groups, with many meeting criteria for Generalised Anxiety Disorder (GAD). GAD has not been reported in previous quantitative studies of food allergy, where measures of anxiety do not necessarily focus specifically on this type of worry. Worry has been reported by mothers taking part in qualitative studies; Springston *et al.* [34] conducted interviews with mothers who described being paralysed with fear and found their worries were constant and overwhelming. GAD in relation to food allergy warrants further investigation.

Three out of the five mothers in the CBT group had mild depression and four scored over the cut-off for clinical caseness on the GHQ12. Few studies have measured depression in parents of children with food allergy but there is evidence that low mood is present in a small proportion of this population [9]. Grief and sorrow was also expressed regarding the loss of having a healthy child, which has only recently been identified in the literature [33].

In comparison to those in the CBT group, the majority of scores in the control group were low or in the normal range and scores generally remained stable across the 12 weeks, which is to be expected if they have developed and continue with adaptive coping strategies. Only a sub-set of parents of children with food allergy may therefore require referral to mental health treatment, a conclusion that has been reached by others [40]. Investigation of the type of parent able to cope compared to those who find it difficult would be beneficial in order to identify possible early interventions that could be put in place to help those parents who might go on to struggle once they have been given a food allergy diagnosis. From the present case series areas identified are: a history of mental illness, a tendency to worry about issues in other areas of their lives, misperceptions about the risk of a severe reaction, complex food allergies which take some time to diagnose or symptoms such as eczema which are difficult to control. It may also be a combination of these factors that is important.

### 4.3. Quality of Life for Parents of Food Allergic Children

Quality of life of parents in the CBT group significantly improved over time. CBT interventions aimed at increasing adaptive coping strategies and confidence in being able to cope with situations such as going on holiday and eating out therefore had a profound effect on reducing the burden felt by parents in these areas.

Most areas of general QoL improved over time, possibly due to parallel improvements in stress, anxiety and depression and an increase in social activities. It is surprising that physical QoL improved in such a short period of time but may be due to improved sleep through a reduction in anxiety, stress and worries.

### 4.4. Limitations of the Study and Areas for Further Research

This study has a number of limitations. As this is a case series only a small number of participants are presented here and a larger sample is needed before preliminary findings from this study can be confirmed. Findings may be due to a response bias with participants who requested CBT being very motivated to make changes. Small numbers meant that demographic differences between the intervention and control participants could not be ascertained and a fully randomised case control study needs to be run in order to ensure these factors are not related to the findings of the study. A study with a larger sample size would also enable analysis of differences regarding age of child, time since diagnosis of food allergy, effect of type of food and symptoms. The present study had a very narrow sample of white British females; CBT with fathers and participants of other socio-economic and ethnic backgrounds is needed, especially as these participants may have very different social and cultural expectations regarding their role in the family. It would also be beneficial to see if CBT is helpful in children themselves.

The significant improvements in participants having CBT may be due to the therapeutic relationship rather than the CBT itself. A good therapeutic relationship is extremely important in producing successful outcomes for clients [41] and this may have produced improvements in cases in the present study, irrespective of the type of therapy they had. CBT interventions appeared to be extremely effective in the present study and could be targeted to specific difficulties discussed by mothers, lending some support to the argument that CBT is effective as a therapy. Studies utilising other therapies or merely providing someone for parents to talk to need to be compared with the efficacy of CBT in order to establish the best type of therapy for this client group. Efficacy of CBT was also only measured at the end of therapy, when a therapeutic relationship was still salient. Longer-term follow up is needed to ascertain lasting effects of CBT.

It is possible that parents would have improved naturally over time without the aid of CBT, however, this is unlikely as not all CBT participants had children who were newly diagnosed and some had been struggling to cope for some time. Comparison with a wait-list control group who meet the criteria to have CBT is needed in order to confirm this. Finally it is not known whether pre-existing mental health difficulties caused parents to develop maladaptive coping strategies and exhibit increased levels of anxiety, worry and depression or whether it was caused by receiving a diagnosis of food allergy for their child. No participants had received therapy for mental health difficulties immediately preceding food allergy diagnosis and not all participants having CBT reported previous mental health difficulties, but this is certainly an area for further investigation with longitudinal research.

### 4.5. Implications of the Results for Clinical Practice

Parents who struggle to manage their child’s allergy and suffer high levels of distress need to be identified early and referred to mental health services when needed. Screening at allergy clinic may help identify those families at risk and results from the present case series offers some areas in which screening may be beneficial, including previous mental health difficulties, high levels of worry and anxiety, lack of social support and negative beliefs surrounding the allergy. Assumptions of risk and hyper-vigilance should also be assessed.

CBT can be expensive, is not always available and there may be long waiting lists so ways in which aspects of CBT could be incorporated into routine clinical visits need to be developed and evaluated. The provision of good quality information about food allergy, which parents can use themselves and provide to their friends, family and child care providers is vitally important. Information about the possible psychological effects of food allergy on parents should be provided, including ways in which parents can develop adaptive coping strategies. The whole family should consider sharing of responsibilities of food allergy to prevent high levels of burden on the mother. For those children that need adrenaline, AAI training should be given at clinic with repeater sessions at regular intervals and all parents should be provided with a trainer AAI to take home so that they and their children can practice.

Provision of information about local and national support groups is important as normalising allergy and fostering an understanding that others have the same difficulties can help to reduce the stigma associated with food allergy and can help prevent parents feeling isolated [37]. Evaluation of the effectiveness of these strategies to reduce distress felt by parents and improve coping is needed.

## 5. Conclusions

Preliminary results from this case series show that CBT may be an effective therapy for parents of children with food allergy in reducing anxiety, worry, stress and depression and improving quality of life. Therapy needs to take into consideration the fact that anxiety may not be completely removed as this may in fact be detrimental to the child’s safety as it may reduce vigilance. Parents need to accept that their child will always live with risk and develop a balance between avoiding that risk and learning to live a relatively normal life [35]. CBT may be a therapy that can aid this learning, improve coping strategies used by parents and reduce the distress they feel.

## Supplementary Files

Supplementary File 1

## Abbreviations

AAI: Adrenaline auto-injector
CBT: Cognitive Behaviour Therapy
FAQL-PB: Food Allergy Quality of Life-Parental Burden questionnaire
GHQ-12: General Health Questionnaire
HADS: Hospital Anxiety and Depression Scale
PSS: Perceived Stress Scale
PWSQ: Penn State Worry Questionnaire
QoL: Quality of Life
WHOQoL-BREF: World Health Organisation Quality of Life Scale

## References

[1] Sicherer S.H. (2011). Epidemiology of food allergy. J. Allergy Clin. Immunol..

[2] Kotz D., Simpson C.R., Sheikh A. (2011). Incidence, prevalence, and trends of general practitioner-recorded diagnosis of peanut allergy in England, 2001 to 2005. J. Allergy Clin. Immunol..

[3] Cummings A., Knibb R.C., King R., Lucas J. (2010). The psychosocial impact of food allergy on children and adolescents: A review. Allergy.

[4] King R.M., Knibb R.C., Hourihane J.O. (2009). Impact of peanut allergy on quality of life, stress and anxiety in the family. Allergy.

[5] Cummings A., Knibb R.C., Erlewyn-Lajeunesse M., King R., Hich G., Lucas J. (2010). Management of nut allergy influences quality of life and anxiety in children and their mothers. Pediatr. Allergy Immunol..

[6] Akeson N., Worth A., Sheikh A. (2007). The psychosocial impact of anaphylaxis on young people and their parents. Clin. Exp. Allergy.

[7] Gillespie C.A., Woodgate R.L., Chalmers K.I., Watson W.T. (2007). “Living with risk”: Mothering a child with food-induced anaphylaxis. J. Pediatr. Nurs..

[8] Boyce J.A., Assa’ad A., Burks A.W., Jones S.M., Sampson H.A., Wood R.A., Plaut M., Cooper S.F., Fenton M.J., Arshad S.H. (2010). Guidelines for the diagnosis and management of food allergy in the United States: Report of the NIAID-Sponsored expert panel. J. Allergy Clin. Immunol..

[9] Knibb R.C., Semper H. (2013). Impact of suspected food allergy on emotional distress and family life of parents prior to allergy diagnosis. Pediatr. Allergy Immunol..

[10] LeBovidge J.S., Timmons K., Rich C. (2008). Evaluation of a group intervention for children with food allergy and their parents. Ann. Allergy Asthma Immunol..

[11] Polloni L., Lazzarotto F., Bonaguro R., Toniolo A., Celegato N., Muraro A. (2014). Psychological care of food allergic children and their families: An exploratory analysis. Pediatr. Allergy Immunol..

[12] Starcevic M. (2006). Anxiety states: A review of conceptual and treatment issues. Curr. Opin. Psychiatry.

[13] Hunot V., Churchill R., Teixeira V., de Lima M. (2007). Psychological therapies for generalised anxiety disorders. Cochrane Database Syst. Rev..

[14] Ross C.J.M., Davis T.M.A., MacDonald G.F. (2005). Cognitive-behavioural treatment combined with asthma education for adults with asthma and coexisting panic disorder. Clin. Nurs. Res..

[15] Coventry P.A., Gellatly J.L. (2008). Improving outcomes for COPD patients with mild-to-moderate anxiety and depression: A systematic review of cognitive behavioural therapy. Br. J. Health Psychol..

[16] Pu C., van den Bergh O., Lemaigre V., Demedts M., Verleden G. (2003). Evaluation of an individualised asthma programme directed at behavioural change. Eur. Respir. J..

[17] Parry G.D., Cooper C.L., Moore J.M., Yadegarfar G., Campbell M.J., Esmonde L., Morice A.H., Hutchcroft B.J. (2010). Cognitive behavioural intervention for adults with anxiety complications of asthma: Prospective randomised trial. Respir. Med..

[18] Wong F.K.D., Poon A. (2010). Cognitive behavioural group treatment for Chinese parents with children with developmental disabilities in Melbourne, Australia: An efficacy study. Aust. N. Z. J. Psychiatry.

[19] American Psychiatric Association (2000). Diagnostic and Statistical Manual of Mental Disorders-Revised.

[20] Zigmond A.S., Snaith R.P. (1983). The hospital anxiety and depression scale. Acta Psychiatr. Scand..

[21] Moorey S., Greer S., Watson M., Gorman C., Rowden L., Tunmore R., Robertson B., Bliss J. (1991). The factor structure and factor stability of the hospital anxiety and depression scale in patients with cancer. Br. J. Psychiatry.

[22] Cohen S., Kamarck T., Mermelstein R. (1983). A global measure of perceived stress. J. Health Soc. Behav..

[23] Cohen S., Williamson G.M. (1988). The Social Psychology of Health.

[24] Cohen B.L., Noone S., Munoz-Furlong A., Sicherer S.H. (2004). Development of a questionnaire to measure quality of life in families with a child with food allergy. J. Allergy Clin. Immunol..

[25] Knibb R.C., Stalker C. (2013). Validation of the food allergy quality of life-parental burden questionnaire in the UK. Qual. Life Res..

[26] Skevington S.M., Lotfy M., O’Connell K.A. (2004). The World Health Organization’s WHOQOL-BREF quality of life assessment: Psychometric properties and results of the international field trial. A report from the WHOQOL Group. Qual. Life Res..

[27] Meyer T.J., Miller M.L., Metzger R.L., Borkovec T.D. (1990). Development and validation of the penn state worry questionnaire. Behav. Res. Ther..

[28] Goldberg D., Williams P. (1988). A User’s Guide to the General Health Questionnaire.

[29] Richards D.A., McDonald B. (1990). Behavioural Psychotherapy: A Handbook for Nurses.

[30] Grant A., Townend M., Mills J., Cockx A. (2008). Assessment and Case Formulation in Cognitive Behavioural Therapy.

[31] Field A. (2013). Discovering Statistics Using IBM SPSS Statistics.

[32] Westbrook D., Kennerley H., Kirk J. (2007). An Introduction to Cognitive Behaviour Therapy: Skills and Applications.

[33] Williams N.A., Parra G.R., Elkin T.D. (2009). Subjective distress and emotional resources in parents of children with food allergy. Child. Health Care.

[34] Springston E.E., Smith B., Shulruff J., Pongracic J., Holl J., Gupta R.S. (2010). Variations in quality of life among caregivers of food allergic children. Ann. Allergy Asthma Immunol..

[35] Herbert L.J., Dahlquist L.M. (2008). Perceived history of anaphylaxis and parental overprotection, autonomy, anxiety and depression in food allergic young adults. J. Clin. Psychol. Med. Settings.

[36] Ravid N.L., Annunziato R.A., Ambrose M.A., Chuang K., Mullarkey C., Sicherer S.H., Shemesh E., Cox A.L. (2012). Mental health and quality-of-life concerns related to the burden of food allergy. Immunol. Allergy Clin. North Am..

[37] Rouf K., White L., Evans K. (2012). A qualitative investigation into the maternal experience of having a young child with severe food allergy. Clin. Child. Psychol. Psychiatry.

[38] Prouessel G., Deschildre A., Castelain C. (2006). Parental knowledge and use of epinephrine auto-injector for children with food allergy. Pediatr. Allergy Immunol..

[39] Umasunthar T., Procktor A., Hodes M., Smith J.G., Gore C., Cox H.E., Marrs T., Hanna H., Phillips K., Pinto C. (2015). Patients’ ability to treat anaphylaxis using adrenaline autoinjectors: A randomised controlled trial. Allergy.

[40] Roy K.M., Roberts M.C. (2011). Peanut allergy in children: Relationships to health-related quality of life, anxiety and parental stress. Clin. Pediatr..

[41] Martin D.J., Gorske J.P., Davis M.K. (2000). Relation of the therapeutic alliance with outcome and other variables: A meta-analytic review. J. Consult. Clin. Psychol..

