# Supplementary material for: Effectiveness of Cognitive Behaviour Therapy for Mothers of Children with Food Allergy: A Case Series
Source: Healthcare (Basel). 2015 Nov 25;3(4):1194–211. doi: 10.3390/healthcare3041194 (PMC4934639; doi:10.3390/healthcare3041194)
Supplement: Supplementary File 1 [file healthcare-03-01194-s001.pdf]

Supplementary Material

Table S1. Mean (standard deviation) and median psychometric scale scores for the CBT and control group, comparing groups at baseline and at 12 weeks.

**Baseline**	**Cases**		**Controls**					
**Scale**	**Mean (SD)**	**Median**	**Mean (SD)**	**Median**	***U***	***Z***	***p***	***r***
**WHOQoL-BREF**								
Physical	3.71 (0.58)	3.17	4.32 (0.35)	4.43	25.5	1.94	0.05	0.58
Psychological	3.36 (0.56)	3.33	4.11 (0.33)	4.17	26.0	2.02	0.05	0.61
Social	3.40 (1.04)	3.33	4.33 (0.60)	4.50	23.0	1.47	0.18	
Environment	3.58 (0.68)	3.13	4.10 (0.33)	4.00	20.0	0.92	0.43	
**FAQL-PB**	86.40 (15.37)	80.00	48.67 (13.10)	51.50	0.00	−2.74	0.004	0.83
**PSS—Stress Scale**	28.80 (9.73)	32.00	18.25 (4.27)	17.00	4.0	−1.48	0.19	
**PSWQ**	56.60 (17.30)	63.00	46.33 (11.69)	48.00	8.5	−1.19	0.25	
**HADS—Anxiety**	11.60 (3.36)	13.00	5.83 (2.32)	6.00	3.0	−2.22	0.03	0.67
**HADS—Depression**	7.00 (3.16)	8.00	1.67 (1.21)	1.50	2.5	−2.30	0.017	0.69
**GHQ-12**	18.40 (7.50)	18.00	8.00 (3.90)	7.50	2.5	−2.29	0.017	0.69
**12 Weeks**	**Cases**		**Controls**					
**Scale**	**Mean (SD)**	**Median**	**Mean (SD)**	**Median**	***U***	***Z***	***p***	***r***
**WHOQoL-BREF**								
Physical	4.26 (0.26)	4.00	4.00 (0.26)	4.00	8.0	−0.49	0.73	
Psychological	3.87 (0.45)	3.83	3.84 (0.56)	3.59	8.0	−0.49	0.73	
Social	3.87 (0.84)	3.67	4.00 (0.61)	4.00	10.0	0.00	1.00	
Environment	4.05 (0.36)	4.13	3.81 (0.58)	3.94	8.0	−0.49	0.73	
**FAQL-PB**	49.60 (11.33)	49.00	57.00 (18.51)	64.00	14.0	0.98	0.41	
**PSS—Stress Scale**	21.00 (8.86)	15.00	25.00 (4.69)	24.50	13.0	0.74	0.56	
**PSWQ**	44.60 (15.49)	38.00	58.25 (9.91)	60.00	16.0	1.47	0.19	
**HADS—Anxiety**	6.40 (2.07)	7.00	7.50 (1.91)	8.00	13.5	0.87	0.41	
**HADS—Depression**	2.40 (1.95)	3.00	4.25 (4.27)	3.00	12.5	0.63	0.56	
**GHQ-12**	5.20 (3.27)	5.00	15.00 (4.16)	15.00	20.0	4.07	0.016	1.23

U = Mann-Whitney *U* test for Independent Samples; *Z* = standardised test statistic, *p* = *p* value for differences between cases and controls, *r* = effect size for significant differences.

Table S2. Mean (standard deviation) and median psychometric scale scores, comparing baseline with end of 12 week scores for the CBT group and for the control group.

Cases	Baseline		12 Weeks					
**Scale**	**Mean (SD)**	**Median**	**Mean (SD)**	**Median**	***T***	***Z***	***p***	***r***
**WHOQoL-BREF**								
Physical	3.71 (0.58)	3.17	4.26 (0.26)	4.00	15.0	2.03	0.042	0.64
Psychological	3.36 (0.56)	3.33	3.87 (0.45)	3.83	14.0	1.76	0.078	
Social	3.40 (1.04)	3.33	3.87 (0.84)	3.67	14.0	1.77	0.077	
Environment	3.58 (0.68)	3.13	4.05 (0.36)	4.13	10.0	1.83	0.068	
**FAQL-PB**	86.40 (15.37)	80.00	49.60 (11.33)	49.00	0.00	−2.02	0.043	0.64
**PSS—Stress Scale**	28.80 (9.73)	32.00	21.00 (8.86)	15.00	1.00	−1.75	0.08	
**PSWQ**	56.60 (17.30)	63.00	44.60 (15.49)	38.00	0.00	−2.02	0.043	0.64
**HADS—Anxiety**	11.60 (3.36)	13.00	6.40 (2.07)	7.00	0.00	−2.03	0.042	0.64
**HADS—Depression**	7.00 (3.16)	8.00	2.40 (1.95)	3.00	0.00	−2.03	0.042	0.64
**GHQ-12**	18.40 (7.50)	18.00	5.20 (3.27)	5.00	0.00	−2.03	0.042	0.64
**Controls**	**Baseline**		**12 Weeks**					
**Scale**	**Mean (SD)**	**Median**	**Mean (SD)**	**Median**	***T***	***Z***	***p***	***r***
**WHOQoL-BREF**								
Physical	4.32 (0.35)	4.43	4.00 (0.26)	4.00	3.0	2.72	0.46	
Psychological	4.11 (0.33)	4.17	3.84 (0.56)	3.59	1.5	−1.29	0.20	
Social	4.33 (0.60)	4.50	4.00 (0.61)	4.00	1.5	−0.82	0.41	
Environment	4.10 (0.33)	4.00	3.81 (0.58)	3.94	0.00	−1.34	0.18	
**FAQL-PB**	48.67 (13.10)	51.50	57.00 (18.51)	64.00	6.0	1.60	0.11	
**PSS—Stress Scale**	18.25 (4.27)	17.00	25.00 (4.69)	24.50	5.0	1.07	0.29	
**PSWQ**	46.33 (11.69)	48.00	58.25 (9.91)	60.00	10.0	1.83	0.068	
**HADS—Anxiety**	5.83 (2.32)	6.00	7.50 (1.91)	8.00	6.0	1.63	0.10	
**HADS—Depression**	1.67 (1.21)	1.50	4.25 (4.27)	3.00	6.0	1.60	0.11	
**GHQ-12**	8.00 (3.90)	7.50	15.00 (4.16)	15.00	10.0	1.83	0.068	

T = Wilcoxon Signed-Ranks Test for Related Samples; *Z* = standardised test statistic, *p* = *p* value for differences between baseline and 12 weeks scores for cases and between baseline and 12 week scores for controls, *r* = effect size for significant differences.

Table S3. Clinical caseness (*n* and %) for worry, anxiety, depression and general mental health for cases and controls at baseline and at 12 weeks.

	Case		Control	
**Scale**	**Baseline (n = 5)**	**12 Weeks (n = 5)**	**Baseline (n = 6)**	**12 Weeks (n = 4)**
Worry (PSWQ)	4 (80.0)	2 (40.0)	3 (50.0)	4 (100.0)
General mental health (GHQ12)	4 (80.0)	0	2 (33.3)	3 (75.0)
Mild anxiety (HADS)	0	2 (40.0)	1 (16.7)	2 (50.0)
Moderate anxiety (HADS)	4 (80.0)	0	0	0
Mild depression (HADS) *	3 (60.0)	0	0	0

* No participant had moderate or severe depression or severe anxiety.

© 2015 by the author; licensee MDPI, Basel, Switzerland. This article is an open access article distributed under the terms and conditions of the Creative Commons Attribution license (http://creativecommons.org/licenses/by/4.0/).
